# Factors Associated With Adherence to Osteoporosis Medications Among Male Veterans

**DOI:** 10.1002/jbm4.10498

**Published:** 2021-06-30

**Authors:** Nicole Sagalla, Richard Lee, Richard Sloane, Kenneth Lyles, Cathleen Colón‐Emeric

**Affiliations:** ^1^ Department of Medicine, Division of Endocrinology Duke University Medical Center Durham NC USA; ^2^ Durham Veterans Affairs Geriatric Research Education and Clinical Center Durham NC USA; ^3^ Department of Medicine, Division of Geriatrics Duke University Medical Center Durham NC USA

**Keywords:** ADHERENCE, HEALTH SERVICES RESEARCH, OSTEOPOROSIS, ANTIRESORPTIVES

## Abstract

Risk factors for nonadherence to osteoporosis medication have been well described for cohorts of women with osteoporosis, but little is known about predictors or mediators of nonadherence in men. We conducted a secondary analysis of a national cohort of male veterans to explore factors associated with nonadherence to osteoporosis medications. We included veterans with a prescription for an oral bisphosphonate or calcitonin between 2000 and 2010. We identified demographic, comorbid, and fracture‐related risk factors by their International Classification of Diseases‐9 (ICD‐9) and Current Procedural Terminology (CPT) codes and used multivariable logistic regression to evaluate their association with adherence. Adherence was measured by medication possession ratio (MPR) over 5 years, starting at the time of their first prescription during the study period and censoring at death or end of study period. Of 135,306 men identified with at least one prescription for an osteoporosis medication during the study period, 90,406 (67%) were nonadherent (MPR < 0.80). The median duration of therapy was 3.2 years (interquartile range [IQR] = 1.7–5.0). In the fully adjusted model, the odds of adherence were lower in those aged <65 years (odds ratio [OR] = 0.87; 95% confidence interval [CI] 0.84–0.89), with no copay (OR = 0.78; 95% CI 0.76–0.80), dementia (OR = 0.87; 95% CI 0.83–0.91), anxiety/depression (OR = 0.92; 95% CI 0.90–0.95), tobacco use (OR = 0.91; 95% CI 0.89–0.94), alcohol abuse (OR = 0.91; 95% CI 0.89–0.94), rheumatoid arthritis (OR = 0.92; 95% CI 0.87–0.97), and on androgen deprivation therapy (OR = 0.89; 95% CI 0.83–0.95). The odds of adherence were higher in whites (OR = 1.14; 95% CI 1.11–1.17), with a prior screening colonoscopy (OR = 1.12; 95% CI 1.09–1.14), on alendronate versus other agents (OR = 1.61; 95% CI 1.55–1.67), with a dual‐energy X‐ray absorptiometry (DXA) (OR = 1.14; 95% CI 1.12–1.17), on glucocorticoids (OR = 1.08; 95% CI 1.02–1.14), and with recent fracture (OR = 1.07; 95% CI 1.04–1.10). In conclusion, adherence to oral bisphosphonates/calcitonin is poor, with particular subgroups at greatest risk. These findings may help tailor approaches for supporting adherence in men prescribed osteoporosis medications. © 2021 The Authors. *JBMR Plus* published by Wiley Periodicals, Inc. on behalf of American Society for Bone and Mineral Research. © 2021 The Authors. *JBMR Plus* published by Wiley Periodicals LLC. on behalf of American Society for Bone and Mineral Research.

## Introduction

In 2010, an estimated 2 million men in the US had osteoporosis and an additional 16.1 million men had low bone mass.^(^ ^1^ ^)^ One in 5 men older than age 50 years sustain an osteoporotic fracture.^(^ ^2^ ^)^ Although women have a higher prevalence of fragility fracture, men have higher fracture‐related mortality.^(^ ^3^ ^)^ However, only a fraction of older men are identified and treated for osteoporosis. Even among the highest‐risk patients who have suffered a hip fracture, only 28.5% are treated for osteoporosis in the following year.^(^ ^4^ ^)^ Furthermore, men and older individuals are less likely to be treated after a hip fracture.^(^ ^4^ ^)^ Despite safe and effective therapy to reduce fracture risk, one‐third to two‐thirds of men are nonadherent to osteoporosis medications at 1 year.^(^ ^5^ ^)^ Studies have shown an inverse relationship between adherence and fracture reduction with fracture rates rising rapidly below a medication possession ratio (MPR; proportion of time covered by the number of dispensed doses) of 0.80.^(^ ^6^, ^7^ ^)^ A systematic review identified men as generally less adherent to osteoporosis therapy than women.^(^ ^8^ ^)^ Although risk factors for nonadherence have been well described for cohorts of women with osteoporosis, little is known about predictors or mediators of nonadherence in men.

To address this knowledge gap, we utilized a large national cohort of male veterans to determine adherence rates and to explore factors associated with nonadherence to osteoporosis medications. We hypothesize that similar risk factors for nonadherence in women will be associated with nonadherence in men.

## Methods

We conducted a secondary analysis of a cohort of male veterans aged 50 to 99 years who received primary care at any of the 146 Veterans Health Administration Medical Centers between 2000 and 2010. The details of this cohort have been described previously.^(^ ^9^, ^10^, ^11^, ^12^ ^)^ Briefly, eligible patients attended at least two primary care visits within 2 consecutive years. Patients with a diagnosis of osteoporosis, fracture, or bisphosphonate use in the 3 years before the start date were excluded. The inpatient, outpatient, fee‐basis, and Pharmacy Benefits Management Services records were merged with Centers for Medicare and Medicaid Services (CMS) Parts A and B claims. The study was approved by the Institutional Review Board (IRB) at the Durham Veterans Affairs (VA) Medical Center.

For this analysis, we selected male veterans with a new diagnosis of osteoporosis during the study period and at least one new prescription for an osteoporosis medication during follow‐up, including alendronate, calcitonin nasal spray, etidronate, and risedronate. Alendronate could be prescribed daily or weekly. Calcitonin and etidronate are dosed daily. Risedronate can be prescribed daily, weekly, or monthly. Patients were excluded if they had at least one prescription for an intravenous or subcutaneous osteoporosis medication because we could not reliably distinguish patients who had these medications prescribed for hypercalcemia or oncologic indications rather than osteoporosis. We identified demographic, comorbid, medication, and fracture‐related risk factors by their International Classification of Diseases‐9 (ICD‐9) and Current Procedural Terminology (CPT) codes. The presence of these covariates was updated at the start of each calendar year and assessed during the year of the initial medication prescription. We evaluated their association with adherence as measured by medication possession ratio (MPR). MPR was defined as the sum of days’ supply dispensed from the start date (the date of the first prescription for osteoporosis medication) to the stop date (5 years after the first prescription, end of the study period, or death of the patient, whichever was earliest) divided by the number of days in that time period. MPR was capped at 1.0. Patients who switched between different osteoporosis therapies had their MPR calculated in the same way, such that they would be considered fully adherent if their dispensed days’ supply of all medications added together was equal to the follow‐up time. Five years was selected as the time horizon because current clinical practice guidelines suggest treating patients for osteoporosis for at least 5 years before reassessing fracture risk and considering a possible drug holiday. This time horizon for calculating MPR therefore gave us a means to identify those who were nonadherent either because they did not take the medication as frequently as prescribed or because they discontinued therapy sooner than recommended.

### Statistical analysis

Descriptive statistics including means, standard deviations, frequencies, and percentages were used to describe the baseline characteristics of the study population. MPR was dichotomized as <0.80 (nonadherent) and ≥0.80 (adherent) based on prior studies demonstrating that fracture rates rise rapidly below this threshold.^(^ ^6^, ^7^ ^)^ We used bivariate analysis to evaluate the association of a predetermined set of candidate risk factor variables with MPR. We then selected clinically and statistically significant factors to be included in the multivariable logistic regression model. A final fully adjusted logistic regression model was conducted containing significant independent variables from the preliminary bivariate analyses.

Assumptions for conducting logistic regression were evaluated. Significance was assessed at *p* = 0.05. SAS v9.4 (SAS Institute, Cary, NC, USA) was used for all analyses.

## Results

Of the 4,338,189 male veterans in the cohort, 135,306 (3.1%) patients had at least one prescription for an osteoporosis medication excluding intravenous and subcutaneous medications. The majority of patients were white (76%) with a mean age of 72.6 years. There were 90,406 patients (66.8%) who were nonadherent with an MPR <0.80 over the 5 years of follow‐up. The mean MPR was 0.544. The median duration of therapy was 3.2 years (interquartile range [IQR] = 1.7–5.0) and the proportion of patients taking at least 3 years of medications was 20.7%. Baseline characteristics stratified by MPR are presented in Table 1.

**Table 1:** Baseline Characteristics of the Cohort

Characteristic			
	MPR <0.80 *n* = 90,406	MPR ≥0.80 *n* = 44,900	Bivariate *p* value
Demographics			
Mean age (SD), years	72.4 (10.2)	73.1 (9.5)	<0.0001
<65 years, *n* (%)	24,697 (27.3%)	10,565 (23.5%))	<0.0001
Race			
White, non‐Hispanic, *n* (%) 102,994 (76.1%)	68,205 (75.4%)	34,789 (77.5%)	<0.0001 (white vs. non‐white)
African American, *n* (%) 10,052 (7.4%)	7408 (8.2%)	2644 (5.9%)	
Unknown, *n* (%) 15,707 (11.6%)	9950 (11.0%)	5757 (12.8%)	
Other, *n* (%) 6553 (4.8%)	4797 (5.3%)	1756 (3.9%)	
Marital status: married vs. other, *n* (%)	58,734 (65.0%)	30,755 (68.5%)	<0.0001
Mean BMI (SD), kg/m^2^	27.2 (4.9)	27.2 (4.8)	0.19
Residence			
Rural, *n* (%)	5757 (6.4%)	2913 (6.5%)	0.40
Homeless, *n* (%)	2068 (2.3%)	708 (1.6%)	<0.0001
Southeast, *n* (%) 51,545 (38.1%)	36,189 (40.0%)	15,356 (34.2%)	<0.0001 (Southeast vs. other US)
Northeast, *n* (%) 23,798 (17.6%)	15,247 (16.9%)	8525 (19.0%)	
Midwest, *n* (%) 29,306 (21.7%)	18,352 (20.3%)	10,954 (24.4%)	
West, *n* (%) 30,657 (22.7%)	20,618 (22.8%)	10,065 (22.4%)	
Medicaid	5180 (5.7%)	3028 (6.7%)	<0.0001
No copay	68,360 (75.6%)	31,211 (69.5%)	<0.0001
Prisoner of war	1098 (1.2%)	601 (1.3%)	0.054
Comorbidities			
Charlson (baseline), mean (SD)	2.10 (2.1)	1.97 (2.0)	<0.0001
Neurologic disease	16,588 (18.4%)	8120 (18.1%)	0.24
Dementia	6634 (7.3%)	2838 (6.3%)	<0.0001
Stroke	1058 (1.2%)	508 (1.1%)	0.52
Parkinson's disease	1980 (2.2%)	827 (1.8%)	<0.0001
Mood disorder (anxiety/depression)	40,360 (44.6%)	18,371 (40.9%)	<0.0001
Other psychiatric disorder	20,189 (22.3%)	9143 (20.4%)	<0.0001
Selective serotonin reuptake inhibitor use	21,565 (23.9%)	9487 (21.1%)	<0.0001
Proton pump inhibitor use	31,288 (34.6%)	15,720 (35.0%)	0.14
Chronic lung disease	34,991 (38.7%)	17,436 (38.8%)	0.64
Chronic liver disease	7022 (7.8%)	3383 (7.5%)	0.13
Tobacco use	25,370 (28.1%)	11,197 (24.9%)	<0.0001
Alcohol abuse	21,046 (23.3%)	9270 (20.7%)	<0.0001
Diabetes, type 1 and 2	28,506 (31.5%)	13,735 (30.6%)	0.0004
Chronic systemic glucocorticoid use	5006 (5.5%)	2477 (5.5%)	0.87
Rheumatoid arthritis	5101 (5.6%)	2338 (5.2%)	0.0009
Prostate cancer	14,851 (16.4%)	7732 (17.2%)	0.0002
Colonoscopy	46,134 (51.0%)	24,362 (54.3%)	<0.0001
Androgen deprivation therapy	3621 (4.0%)	1653 (3.7%)	0.004
Non‐skin cancer	31,655 (35.0%)	16,607 (37.0%)	<0.0001
Congestive heart failure	9091 (10.1%)	4546 (10.1%)	0.69
CKD diagnosis code	30,321 (33.5%)	14,799 (33.0%)	0.033
CKD‐eGFR, mean (SD)	45.6 (50.0)	45.4 (50.0)	0.58
Endocrine disorder	3367 (3.7%)	1630 (3.6%)	0.39
Traditional antiepileptic drug use	11,983 (13.3%)	6300 (14.0%)	<0.0001
Medication/therapy			
Medication			
Alendronate	75,694 (83.7%)	40,127 (89.4%)	<0.0001 (alendronate vs. others)
Etidronate	438 (0.5%)	85 (0.2%)	
Risedronate	5139 (5.7%)	2175 (4.8%)	
Calcitonin	9064 (10.0%)	2475 (5.5%)	
Received DXA during follow‐up	45,808 (50.7%)	24,410 (54.4%)	<0.0001
FRAX‐without BMD (major), mean (SD)	6.29 (3.24)	6.33 (3.15)	0.02
Fracture^a^ within 1 year of starting medication (yes/no)	13,820 (15.3%)	7148 (15.9%)	0.02
Number of PCP visits/year, mean (SD)	7.7 (7.2)	7.8 (7.1)	0.0009

BMI = body mass index; CKD = chronic kidney disease; eGFR = estimated glomerular filtration rate; DXA = dual‐energy X‐ray absorptiometry; BMD = bone mineral density; PCP = primary care physician.

^a^ Fracture includes any pelvic, hip, femur, humerus, vertebral, forearm fracture.

### Associations with medication adherence

We evaluated the association of demographic variables, comorbidities, health care utilization, and osteoporosis‐related variables with adherence. Among the demographic variables, the odds of adherence were lower in those aged <65 years versus ≥65 years (odds ratio [OR] = 0.87; 95% confidence interval [CI] 0.84–0.89) and with no copay (OR = 0.78; 95% CI 0.76–0.80). The odds of adherence were higher in whites versus non‐whites (OR = 1.14; 95% CI 1.11–1.17).

Of the comorbidities assessed, adherence was not associated with the Charlson comorbidity index but was associated with the following comorbidities: the odds of adherence were lower in those with dementia (OR = 0.87; 95% CI 0.83–0.91), anxiety/depression (OR = 0.92; 95% CI 0.90–0.95), tobacco use (OR = 0.91; 95% CI 0.89–0.94), alcohol abuse (OR = 0.91; 95% CI 0.89–0.94), and rheumatoid arthritis (OR = 0.92; 95% CI 0.87–0.97). For medications associated with bone loss, those on androgen deprivation therapy had lower odds of adherence (OR = 0.89; 95% CI 0.83–0.95), whereas those prescribed glucocorticoids had higher odds of adherence (OR = 1.08; 95% CI 1.02–1.14). In terms of health care utilization, the odds of adherence were higher for those who had a prior screening colonoscopy (OR = 1.12; 95% CI 1.09–1.14) (Table 2).

**Table 2:** Odds Ratios (OR) and 95% Confidence Intervals (CIs) for the Association Between Patient Factors and Osteoporosis Medication Adherence

	OR	95% CI
Age < 65 years	0.87	(0.84–0.89)
White race	1.14	(1.11–1.17)
No copay	0.78	(0.76–0.80)
Charlson score	0.99	(0.98–1.00)
Dementia	0.87	(0.83–0.91)
Psychiatric disease	1.01	(0.98–1.04)
Anxiety/depression	0.92	(0.90–0.95)
Tobacco use	0.91	(0.89–0.94)
Alcohol abuse	0.91	(0.89–0.94)
Diabetes	0.98	(0.95–1.00)
Colonoscopy	1.12	(1.09–1.14)
Alendronate	1.61	(1.55–1.67)
Prior DXA	1.14	(1.12–1.17)
Glucocorticoid	1.08	(1.02–1.14)
Rheumatoid arthritis	0.92	(0.87–0.97)
Prostate cancer	1.05	(1.01–1.09)
ADT	0.89	(0.83–0.95)
Fracture within 1 year of starting medication	1.07	(1.04–1.10)

DXA = dual‐energy X‐ray absorptiometry; ADT = androgen deprivation therapy.

For osteoporosis‐related factors, the odds of adherence were higher in those who had a dual‐energy X‐ray absorptiometry (DXA) during follow‐up (OR = 1.14; 95% CI 1.12–1.17) and a fracture within 1 year before or after starting osteoporosis medication (OR 1.07; 95% CI 1.04–1.10). Fractures included any pelvic, hip, femur, humerus, vertebral, or forearm fracture. Those prescribed alendronate had higher odds of adherence than those prescribed other osteoporosis therapies (OR = 1.61; 95% CI 1.55–1.67).

## Discussion

One‐fifth of all men aged 50 years and older in the US are potentially eligible for osteoporosis treatment, according to 2008 National Osteoporosis Foundation (NOF) thresholds, either because of a prior fragility hip or vertebral fracture, osteoporosis by DXA, or osteopenia with elevated fracture risk attributable to other clinical risk factors.^(^ ^13^ ^)^ Among the few that are identified and initiated on osteoporosis treatment, a large proportion do not take the medication as prescribed or discontinue the medication altogether.^(^ ^5^ ^)^ In this large cohort study of male veterans, less than one‐third of patients were adherent with a 5‐year course for oral bisphosphonates, with nearly 80% discontinuing within 3 years. These numbers are comparable to prior studies in women; a systematic review identified 40% to 85% of women were adherent at 1 year and 26% to 72% had discontinued their medication by 1 year.^(^ ^14^ ^)^ One study evaluated adherence over 5 years and found 23% of women (*n* = 111) had an annual adherence of MPR ≥ 0.80.^(^ ^15^ ^)^ However, it may be difficult to compare across studies depending on the time horizon or censoring strategy because there are several accepted ways of calculating MPR.^(^ ^16^ ^)^ We chose a 5‐year time horizon to assess whether patients were receiving the guideline concordant treatment duration for oral bisphosphonates.

Interventions such Fracture Liaison Services have been employed to improve osteoporosis medication adherence and have demonstrated decreased fracture rates.^(^ ^17^, ^18^, ^19^ ^)^ To best allocate resources within a large health system, identification of those at greatest risk for nonadherence is imperative so that they can be targeted for these types of interventions. Our study identified a number of key subgroups at high risk for nonadherence, focused on demographic, comorbidity, and health care utilization factors.

Of the demographic variables, our study is consistent with multiple prior studies showing worse adherence among non‐white and younger patients.^(^ ^8^ ^)^ While higher cost of medications has previously been shown to be associated with medication discontinuation,^(^ ^20^ ^)^ our study found that patients who had no copay had higher odds of poor adherence. At the VA, those with no copay are likely of low socioeconomic status. It may follow that those of low socioeconomic status are at higher risk of nonadherence.

Regarding comorbidities assessed, our study is consistent with other studies showing poorer adherence among those with tobacco use, alcohol abuse, autoimmune disease including rheumatoid arthritis, and psychiatric conditions including depression.^(^ ^8^ ^)^ Whereas prior studies have been equivocal, our study showed significant nonadherence among those with dementia. Also, consistent with prior studies showing better adherence among those with a history of fracture, those with a fracture within 1 year of starting osteoporosis medication in our study had better adherence. Finally, to our knowledge, androgen deprivation therapy has not been previously shown to be associated with nonadherence.

In terms of health care utilization, several studies have shown improved adherence with prior osteoporosis screening such as with DXA.^(^ ^21^, ^22^, ^23^, ^24^ ^)^ More studies have shown poorer adherence with calcitonin than other medications, but regarding different preparations of bisphosphonates, results were mixed.^(^ ^8^ ^)^ Having a screening colonoscopy or using glucocorticoids have not been evaluated as factors associated with adherence previously. Patients who have had a screening colonoscopy may represent those who are more adherent to medical recommendations or screening interventions in general.

A major strength of this study was the large size and national scope of the cohort focused on older men. A recent systematic review identified two small studies focusing on men among 124 studies evaluating factors associated with adherence.^(^ ^8^ ^)^ In one of the studies of 198 veterans at the Madison, WI, VA, younger age and tobacco use were associated with nonadherence to alendronate and bone mineral density (BMD) testing was associated with adherence.^(^ ^25^ ^)^ In the second study of 333 men from Taiwan, those with rheumatoid arthritis or baseline BMD testing were less likely to have poor adherence.^(^ ^26^ ^)^

However, because the current analysis was based on a population of veterans, it may not be generalizable to other populations. The main limitation of this study was the use of administrative and claims data to assess adherence and comorbidities, which limits which factors can be evaluated. There is also a lack of sensitivity for ICD‐9 codes for many chronic conditions, and there is a high proportion of missing data for race and marital status. To mitigate these factors, we used validated ICD‐9 code algorithms combined with medication prescriptions to improve sensitivity and specificity. Rather than impute missing demographic data, we used a “missing” category to avoid misclassification bias. We were unable to exclude patients who may have been taking a bisphosphonate for Paget's disease. We were unable to assess medications prescribed outside the VA, but veterans enrolled in primary care receive >90% of their chronic disease management medications from the VA pharmacy.

The results from the current study will inform patient‐centered interventions targeted toward those at greatest risk for nonadherence. For example, because younger patients may have a lack of perceived benefit of therapy, an educational intervention may improve adherence. Those with dementia may have trouble remembering to take medications, and a reminder such as a phone call, text message, or alarm clock may improve adherence. Although many interventions have been studied across a general population with mixed results,^(^ ^18^, ^19^ ^)^ future studies should focus on specific subgroups and tailor interventions to address specific barriers to adherence.

Adherence to oral bisphosphonates and calcitonin for osteoporosis/osteopenia is poor, with two‐thirds of veterans in our national sample being nonadherent by MPR. Certain factors were found to be associated with nonadherence, including younger age, non‐white race, no copay, lack of screening colonoscopy or DXA, prescription for calcitonin and oral bisphosphonates other than alendronate, dementia, anxiety/depression, rheumatoid arthritis, tobacco or alcohol use, androgen deprivation therapy, and no recent fracture. These findings may help tailor approaches for supporting adherence in men prescribed certain osteoporosis medications.

## Disclosures

KL is a Trustee of the National Osteoporosis Foundation, a consultant for Health Stream, Viking, and founder and equity owner of Faculty Connection, LLC and BisCardia, Inc. All other authors state that they have no conflicts of interest.

## Author Contributions

**Nicole Sagalla:** Conceptualization; data curation; formal analysis; investigation; methodology; writing‐original draft; writing‐review & editing. **Richard Lee:** Conceptualization; data curation; formal analysis; investigation; methodology; supervision; writing‐original draft; writing‐review & editing. **Richard Sloane:** Conceptualization; data curation; formal analysis; methodology; resources; software; writing‐review & editing. **Kenneth Lyles:** Conceptualization; data curation; formal analysis; methodology; supervision; writing‐review & editing. **Cathleen Colón‐Emeric:** Conceptualization; data curation; formal analysis; funding acquisition; methodology; supervision; writing‐original draft; writing‐review & editing.

### Peer Review

The peer review history for this article is available at https://publons.com/publon/10.1002/jbm4.10498.

## References

[1] Wright NC , Looker AC , Saag KG , et al. The recent prevalence of osteoporosis and low bone mass in the United States based on bone mineral density at the femoral neck or lumbar spine. J Bone Miner Res. 2014;29(11):2520‐2526. 24771492 10.1002/jbmr.2269 PMC4757905

[2] Melton LJ 3rd , Atkinson EJ , O'Connor MK , O'Fallon WM , Riggs BL . Bone density and fracture risk in men. J Bone Miner Res. 1998;13(12):1915‐1923. 9844110 10.1359/jbmr.1998.13.12.1915

[3] Center JR , Nguyen TV , Schneider D , Sambrook PN , Eisman JA . Mortality after all major types of osteoporotic fracture in men and women: an observational study. Lancet. 1999;353(9156):878‐882. 10093980 10.1016/S0140-6736(98)09075-8

[4] Solomon DH , Johnston SS , Boytsov NN , McMorrow D , Lane JM , Krohn KD . Osteoporosis medication use after hip fracture in U.S. patients between 2002 and 2011. J Bone Miner Res. 2014;29(9):1929‐1937. 24535775 10.1002/jbmr.2202 PMC4258070

[5] Mikyas Y , Agodoa I , Yurgin N . A systematic review of osteoporosis medication adherence and osteoporosis‐related fracture costs in men. Appl Health Econ Health Policy. 2014;12(3):267‐277. 24477429 10.1007/s40258-013-0078-1

[6] Patrick AR , Brookhart MA , Losina E , et al. The complex relation between bisphosphonate adherence and fracture reduction. J Clin Endocrinol Metab. 2010;95(7):3251‐3259. 20444916 10.1210/jc.2009-2778 PMC2928897

[7] Siris ES , Harris ST , Rosen CJ , et al. Adherence to bisphosphonate therapy and fracture rates in osteoporotic women: relationship to vertebral and nonvertebral fractures from 2 US claims databases. Mayo Clin Proc. 2006;81(8):1013‐1022. 16901023 10.4065/81.8.1013

[8] Yeam CT , Chia S , Tan HC , Kwan YH , Fong W , Seng JJ . A systematic review of factors affecting medication adherence among patients with osteoporosis. Osteoporos Int. 2018;29(12):2623‐2637. 30417253 10.1007/s00198-018-4759-3

[9] Colon‐Emeric CS , Pieper CF , van Houtven CH , et al. Limited osteoporosis screening effectiveness due to low treatment rates in a national sample of older men. Mayo Clin Proc. 2018;93(12):1749‐1759. 30497697 10.1016/j.mayocp.2018.06.024 PMC6338211

[10] Hall RK , Sloane R , Pieper C , et al. Competing risks of fracture and death in older adults with chronic kidney disease. J Am Geriatr Soc. 2018;66(3):532‐538. 29319880 10.1111/jgs.15256 PMC5849495

[11] Lee RH , Sloane R , Pieper C , et al. Clinical fractures among older men with diabetes are mediated by diabetic complications. J Clin Endocrinol Metab. 2018;103(1):281‐287. 29099931 10.1210/jc.2017-01593 PMC5761492

[12] Ogunwale AN , Colon‐Emeric CS , Sloane R , Adler RA , Lyles KW , Lee RH . Acetylcholinesterase inhibitors are associated with reduced fracture risk among older veterans with dementia. J Bone Miner Res. 2020;35(3):440‐445. 31711264 10.1002/jbmr.3916 PMC7215241

[13] Dawson‐Hughes B , Looker AC , Tosteson AN , Johansson H , Kanis JA , Melton LJ . The potential impact of new National Osteoporosis Foundation guidance on treatment patterns. Osteoporos Int. 2010;21(1):41‐52. 19705046 10.1007/s00198-009-1034-7

[14] Fardellone P , Lello S , Cano A , et al. Real‐world adherence and persistence with bisphosphonate therapy in postmenopausal women: a systematic review. Clin Ther. 2019;41(8):1576‐1588. 31151814 10.1016/j.clinthera.2019.05.001

[15] Dugard MN , Jones TJ , Davie MW . Uptake of treatment for osteoporosis and compliance after bone density measurement in the community. J Epidemiol Community Health. 2010;64(6):518‐522. 19679704 10.1136/jech.2008.084558

[16] Raebel MA , Schmittdiel J , Karter AJ , Konieczny JL , Steiner JF . Standardizing terminology and definitions of medication adherence and persistence in research employing electronic databases. Med Care. 2013;51(8 Suppl 3):S11‐S21. 23774515 10.1097/MLR.0b013e31829b1d2a PMC3727405

[17] Wu CH , Tu ST , Chang YF , et al. Fracture liaison services improve outcomes of patients with osteoporosis‐related fractures: a systematic literature review and meta‐analysis. Bone. 2018;111:92‐100. 29555309 10.1016/j.bone.2018.03.018

[18] Jaleel A , Saag KG , Danila MI . Improving drug adherence in osteoporosis: an update on more recent studies. Ther Adv Musculoskelet Dis. 2018;10(7):141‐149. 30023009 10.1177/1759720X18785539 PMC6047244

[19] Hiligsmann M , Salas M , Hughes DA , et al. Interventions to improve osteoporosis medication adherence and persistence: a systematic review and literature appraisal by the ISPOR Medication Adherence & Persistence Special Interest Group. Osteoporos Int. 2013;24(12):2907‐2918. 23636230 10.1007/s00198-013-2364-z

[20] Yun H , Curtis JR , Guo L , et al. Patterns and predictors of osteoporosis medication discontinuation and switching among Medicare beneficiaries. BMC Musculoskelet Disord. 2014;15:112. 24684864 10.1186/1471-2474-15-112 PMC4022369

[21] Yu SF , Yang TS , Chiu WC , et al. Nonadherence to anti‐osteoporotic medications in Taiwan: physician specialty makes a difference. J Bone Miner Metab. 2013;31(3):351‐359. 23377623 10.1007/s00774-013-0424-2

[22] Solomon DH , Avorn J , Katz JN , et al. Compliance with osteoporosis medications. Arch Intern Med. 2005;165(20):2414‐2419. 16287772 10.1001/archinte.165.20.2414

[23] Lo JC , Pressman AR , Omar MA , Ettinger B . Persistence with weekly alendronate therapy among postmenopausal women. Osteoporos Int. 2006;17(6):922‐928. 16609824 10.1007/s00198-006-0085-2

[24] Cotte FE , Fardellone P , Mercier F , Gaudin AF , Roux C . Adherence to monthly and weekly oral bisphosphonates in women with osteoporosis. Osteoporos Int. 2010;21(1):145‐155. 19459025 10.1007/s00198-009-0930-1 PMC2788149

[25] Hansen KE , Swenson ED , Baltz B , Schuna AA , Jones AN , Elliott ME . Adherence to alendronate in male veterans. Osteoporos Int. 2008;19(3):349‐356. 17898921 10.1007/s00198-007-0471-4

[26] Chiu CK , Kuo MC , Yu SF , Su BY , Cheng TT . Adherence to osteoporosis regimens among men and analysis of risk factors of poor compliance: a 2‐year analytical review. BMC Musculoskelet Disord. 2013;14:276. 24060442 10.1186/1471-2474-14-276 PMC3849144

